# Creatinine- and cystatin C-based estimated glomerular filtration rate slopes for the prediction of kidney outcome: a comparative retrospective study

**DOI:** 10.1186/s12882-019-1403-1

**Published:** 2019-06-11

**Authors:** Suhyun Kim, Subin Hwang, Hye Ryoun Jang, Insuk Sohn, Hyeon Seon Ahn, Hyung-Doo Park, Wooseong Huh, Dong-Chan Jin, Yoon-Goo Kim, Dae Joong Kim, Ha Young Oh, Jung Eun Lee

**Affiliations:** 1 Division of Nephrology, Department of Internal Medicine, Samsung Medical Center, Sungkyunkwan University School of Medicine, 81 Irwon-ro, Gangnam-gu, 06351 Seoul, Republic of Korea; 2 Department of Internal Medicine, Seoul Paik Hospital, Inje University College of Medicine, Seoul, Republic of Korea; 3 Statistics and Data Center, Samsung Medical Center, Seoul, Republic of Korea; 4 Department of Laboratory Medicine and Genetics, Samsung Medical Center, Sungkyunkwan University School of Medicine, Seoul, Republic of Korea; 5 Department of Internal Medicine, College of Medicine, The Catholic University of Korea, Seoul, Republic of Korea

**Keywords:** Chronic kidney disease (CKD), Estimated glomerular filtration rate (eGFR), GFR slope, Disease progression, Serum creatinine, Serum cystatin C, Renal outcome

## Abstract

**Background:**

Many studies have evaluated the usefulness of creatinine- (eGFRcr) and cystatin C-based estimated glomerular filtration rate (eGFRcys) at specific time points in predicting renal outcome. This study compared the performance of both eGFR changing slopes in identifying patients at high risk of end-stage renal disease (ESRD).

**Methods:**

From 2012 to 2017, patients with more than three simultaneous measurements of serum creatinine and cystatin C for 1 year were identified. Rapid progression was defined as eGFR slope < − 5 mL/min/1.73 m^2^/year. The primary outcome was progression to ESRD.

**Results:**

Overall, 1323 patients were included. The baseline eGFRcr and eGFRcys were 39 (27–48) and 38 (27–50) mL/min/1.73 m^2^, respectively. Over 2.9 years (range, 2.0–3.8 years) of follow-up, 134 subjects (10%) progressed to ESRD. Both the eGFRcr and eGFRcys slopes were associated with a higher risk of ESRD, independently of baseline eGFR (hazard ratio [HR] = 0.986 [0.982–0.991] and HR = 0.988 [0.983–0.993], respectively; all *p* <  0.001). The creatinine- and cystatin C-based rapid progressions were associated with increased risk of ESRD (HR = 2.22 [1.57–3.13], HR = 2.03 [1.44–2.86], respectively; all *p* <  0.001). In the subgroup analyses, the rapid progression group, defined on the basis of creatinine levels (*n* = 503), showed no association between the eGFRcys slope and ESRD risk (*p* = 0.31), whereas the eGFRcr slope contributed to further discriminating higher ESRD risk in the subjects with rapid progression based on eGFRcys slopes (*n* = 463; *p* = 0.003).

**Conclusions:**

Both eGFR slopes were associated with future ESRD risk. The eGFRcr slope was comparable with the eGFRcys slope in predicting kidney outcome.

## Background

Chronic kidney disease (CKD) is a major public health problem worldwide and is associated with adverse outcome and high medical costs [1, 2]. It is important to identify the high risk of CKD progression to slow the worsening of kidney dysfunction and to prepare for the transition toward end-stage renal disease (ESRD) [3, 4]. Many studies have shown that early, short-term changes in glomerular filtration rate (GFR) can predict CKD progression [5–7]. Current clinical guidelines recommend assessing the risk of ESRD by monitoring the estimated GFR (eGFR) trends over time [8–11].

The eGFR is a practical method for assessment of kidney function [11, 12]. Serum creatinine level is a standard marker extensively used to calculate eGFR [13, 14]. However, serum creatinine levels are affected by muscle mass, age, sex, race, and diet besides kidney function [15, 16]. Serum cystatin C concentration has been introduced as an endogenous marker of kidney function and has been used to calculate the eGFR. It is reported to be independent of age, sex, and body mass [17, 18]. Several studies have demonstrated similar accuracies between creatinine-(eGFRcr) and cystatin C-based eGFRs (eGFRcys) in the assessment of static kidney function [19, 20]. The advantage of the cystatin C-based equation is that it is independent of race and obtains a more accurate estimation of GFR in patients with muscle wasting or chronic illness [21].

Nonetheless, data on the comparative usefulness of serum cystatin C and serum creatinine levels for the assessment of eGFR trends over time are limited. In this study, we calculated eGFRcr and eGFRcys slopes by using serial, simultaneous measurements of both markers over a 1-year period. We compared the prognostic power for progression to ESRD of both eGFRcr and eGFRcys slopes in patients with CKD.

## Methods

### Study population

For the present study, we retrospectively identified 1555 adults (aged ≥18 years) with CKD who had undergone at least three simultaneous measurements of both creatinine and cystatin C levels over a 1-year period at an outpatient nephrology clinic of a 2000-bed tertiary hospital between October 2012 and May 2017. We set the baseline as the first simultaneous measurement. CKD was defined on the basis of a baseline eGFR of < 60 mL/min/1.73 m^2^. Each CKD stage represents a level of kidney function as defined by a GFR, as follows: stage 3, eGFR 30–59 mL/min/1.73 m^2^; stage 4, eGFR 15–29 mL/min/1.73 m^2^; stage 5, eGFR < 15 mL/min/1.73 m^2^*.*We excluded 203 participants because they were treated with dialysis or a kidney transplant, or were lost to follow-up within 1 year from baseline. Twenty-nine participants were excluded because they lacked three serial measurements within 1 year. Finally, 1323 patients with non-dialysis CKD stage 3–5 were analyzed in the study.

The study protocol was approved by the institutional review board (IRB) of Samsung Medical Center. We did not obtain written informed consent from the participants for this retrospective study because patient information was anonymized and deidentified prior to analysis. The IRB ensured the anonymity of the data and approved this consent procedure.

### Procedures

Demographic and clinical data at baseline were collected from medical records and included age, sex, height, weight, body mass index (BMI), etiology of CKD, comorbidities, and medication history. Baseline was defined as the time at which the first serum creatinine and cystatin-C levels were measured simultaneously at enrollment. The laboratory test values measured within 3 months from this point were considered baseline data. Laboratory data included serum albumin levels; baseline and serial measurements of creatinine and cystatin C levels; protein-to-creatinine ratio (PCR). We defined patients with diabetes as those with hemoglobin A1c levels of ≥6.5%; self-reported use of a glucose-lowering agent, including insulin; or self-reported incidence of diabetes. Patients were defined as having hypertension if they were receiving antihypertensive medication or self-reported its incidence.

The eGFRs were calculated using the Chronic Kidney Disease Epidemiology Collaboration (CKD-EPI) 2009 creatinine equation, the CKD-EPI 2012 cystatin C equation, and the creatinine-cystatin C equation [22]. Baseline eGFR values were calculated using the CKD-EPI creatinine-cystatin C equation (eGFRcr-cys). The eGFR slopes were determined as an annual eGFR change calculated from a linear regression model by using all simultaneous measurements of serum creatinine and cystatin C levels over a 1-year period. We tested two indexes of eGFR slope, namely the eGFRcr and eGFRcys slopes during the first year. The number of median eGFRs used for the calculation of slopes was five (four to six) for each slope. We defined rapid progression as an eGFR slope steeper than − 5 mL/min/1.73 m^2^/year [11].

Serum creatinine level was measured using the Roche Hitachi-D-Module instrument with the Roche Creatinine Plus assay, which is traceable to the National institute of Standards and Technology creatinine standard reference material (SRM 914) [23]. Serum cystatin C level was measured using a particle-enhanced immunoassay (Gentian, Moss, Norway), traceable to the international reference material: ERM-DA471/IFCC, using an Architect ci8200 analyzer.

The primary outcome was progression to ESRD during the follow-up period. We defined ESRD as the initiation of renal replacement therapy (i.e., hemodialysis or peritoneal dialysis) or receipt of kidney transplantation. Outcomes from October 2013 through May 2017 were retrieved from the ESRD registry of the Korean Society of Nephrology [24].

### Statistical analyses

Descriptive data were reported as mean ± standard deviation (SD) for continuous and normally distributed variables, median and interquartile range (IQR) for non-normally distributed continuous variables, and numbers (percentage) for categorical variables. The 1-year eGFR slope values were calculated using linear regression analyses. The cumulative risk of ESRD was estimated using the Kaplan-Meier method. The 3-year progression rate was calculated using the Kaplan-Meier product-limit method and compared using the log-rank test. Cox proportional hazard models were used to estimate the association of the eGFR slope with the risk of ESRD adjusted for the baseline eGFRcr-cys. The significance of the association between predictor and outcome was expressed as a hazard ratio (HR) with 95% confidence intervals (CI). The C-index was used to assess discrimination for each of the two eGFR markers*.* Statistical analyses were performed using SAS version 9.4 (SAS Institute, Cary, NC) and R software version 3.4.0 (R Project for Statistical Computing). A *p* value of < 0.05 was considered statistically significant.

## Results

### Characteristics of the study participants

The baseline characteristics of the study population (*n* = 1323) are presented in Table 1. The patients’ median age was 56 years (45–68 years), and 473 (36%) of the patients were female. Overall, 449 patients (34%) had diabetes and 675 (51%) received an angiotensin-converting enzyme inhibitor (ACE-I) or angiotensin receptor blocker (ARB). The eGFRcr, eGFRcys, and eGFRcr-cys values (mL/min/1.73 m^2^) were 39 (27–48), 38 (27–50), and 38 (27–49), respectively.

**Table 1:** Baseline characteristics of the study subjects

Variable	*n* = 1323
Age, median (IQR), years	56 (45–68)
Female, *n* (%)	473 (36)
Body mass index, median (IQR), kg/m^2^	23.9 (21.7–26.3)
Diabetes, *n* (%)	449 (34)
Hypertension, *n* (%)	1165 (88)
Malignancy, *n* (%)	202 (15)
Kidney transplantation, *n* (%)	238 (18)
ACE-I or ARB treatment, *n* (%)	675 (51)
Spironolactone treatment, *n* (%)	41 (3)
CKD stage^a^, *n* (%) 3a/3b/4/5	450 (34)/471 (36)/337 (25)/65 (5)
Creatinine, median (IQR), mg/dL eGFRcr, median (IQR), mL/min/1.73 m^2^	1.8 (1.5–2.3) 39 (27–48)
Cystatin C, median (IQR), mg/L eGFRcys, median (IQR), mL/min/1.73 m^2^	1.7 (1.4–2.2) 38 (27–50)
eGFRcr-cys, median (IQR), mL/min/1.73 m^2^	38 (27–49)
Serum albumin, median (IQR), g/dL	4.3 (4.0–4.5)
PCR, median (IQR), μg/mg Cr	0.50 (0.16–1.33)

Values are expressed as median (interquartile range) or percentage, as appropriate

*ACE-I* Angiotensin-converting enzyme inhibitor, *ARB* Angiotensin receptor blocker, *CKD* Chronic kidney disease, *KT* Kidney transplantation, *GN* Glomerulonephritis, *DM* Diabetes mellitus, *PCKD* Polycystic kidney disease, *eGFR* Estimated glomerular filtration rate, *eGFRcr* Creatinine-based eGFR, *eGFRcys* Cystatin C-based eGFR

^a^CKD stages were categorized according to baseline eGFRcr-cys values

**Fig. 1:**
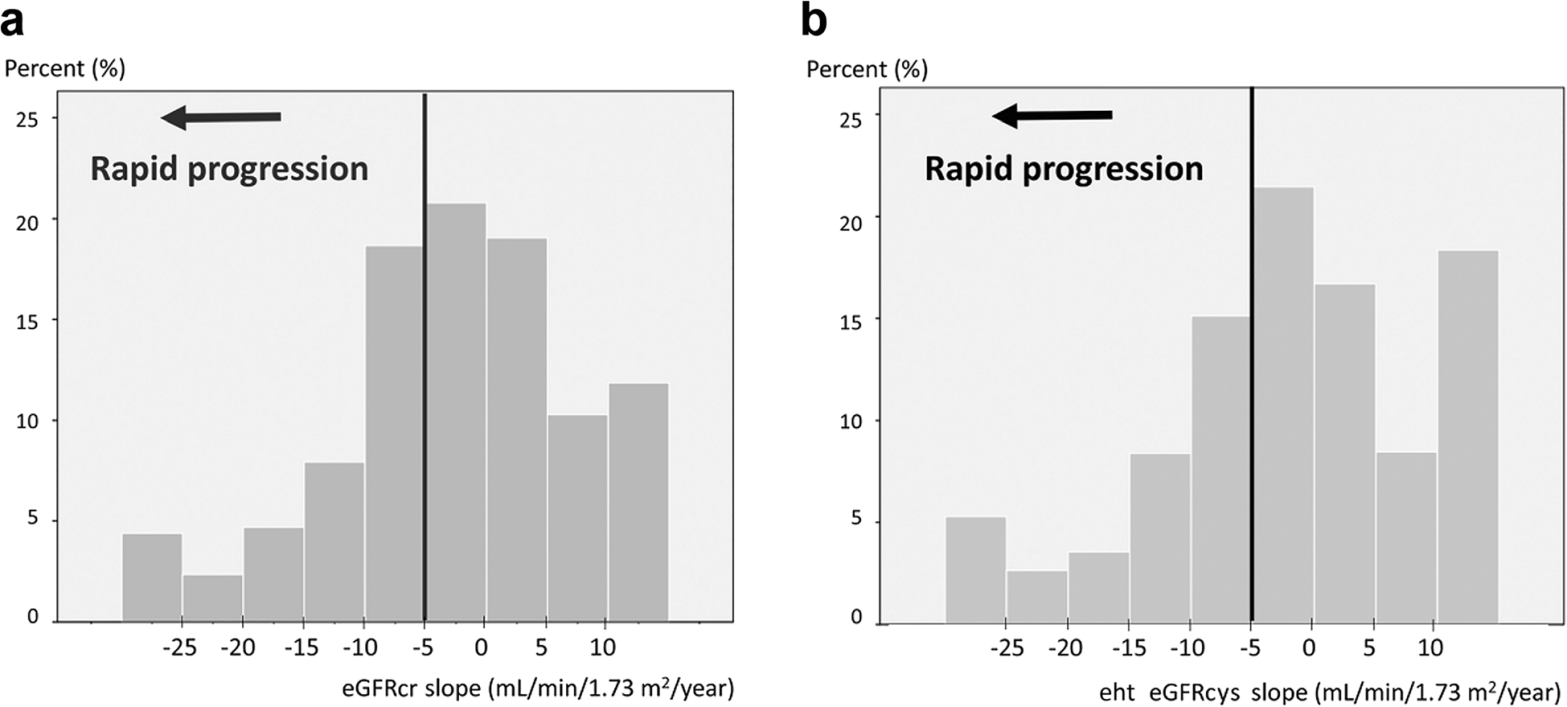
Distributions of the 1-year estimated glomerular filtration rate slopes. The median value of the eGFRcr slope was − 1.83 mL/min/1.73 m^2^/year (interquartile range [IQR]: − 8.39 to 3.86 mL/min/1.73 m^2^/year) (**a**). Of the subjects enrolled in the study (*n* = 1323), 503 (38%) met the criteria for rapid progression based on the eGFRcr slope. The median value of the eGFRcys slope was − 1.73 mL/min/1.73 m^2^/year (IQR: − 7.98 to 6.07 mL/min/1.73 m^2^/year), and 463 (35%) subjects met the criteria for rapid progression based on the eGFRcys slope (**b**). eGFR, estimated glomerular filtration rate; eGFRcr, creatinine-based eGFR; eGFRcys, cystatin C-based eGFR

The 1-year eGFRcr and eGFRcys slopes were − 1.83 mL/min/1.73 m^2^/year (− 8.39 to 3.86 mL/min/1.73 m^2^/year) and − 1.73 mL/min/1.73 m^2^/year (− 7.98 to 6.07 mL/min/1.73 m^2^/year), respectively. When we categorized the participants with eGFR slopes steeper than − 5 mL/min/1.73 m^2^/year into the rapid progression group, 503 (38%) and 463 subjects (35%) met the criteria for rapid progression as defined on the basis of the eGFRcr and eGFRcys slopes (Fig. 1).

### One-year estimated glomerular filtration rate slope and the risk of end-stage renal disease

**Fig. 2:**
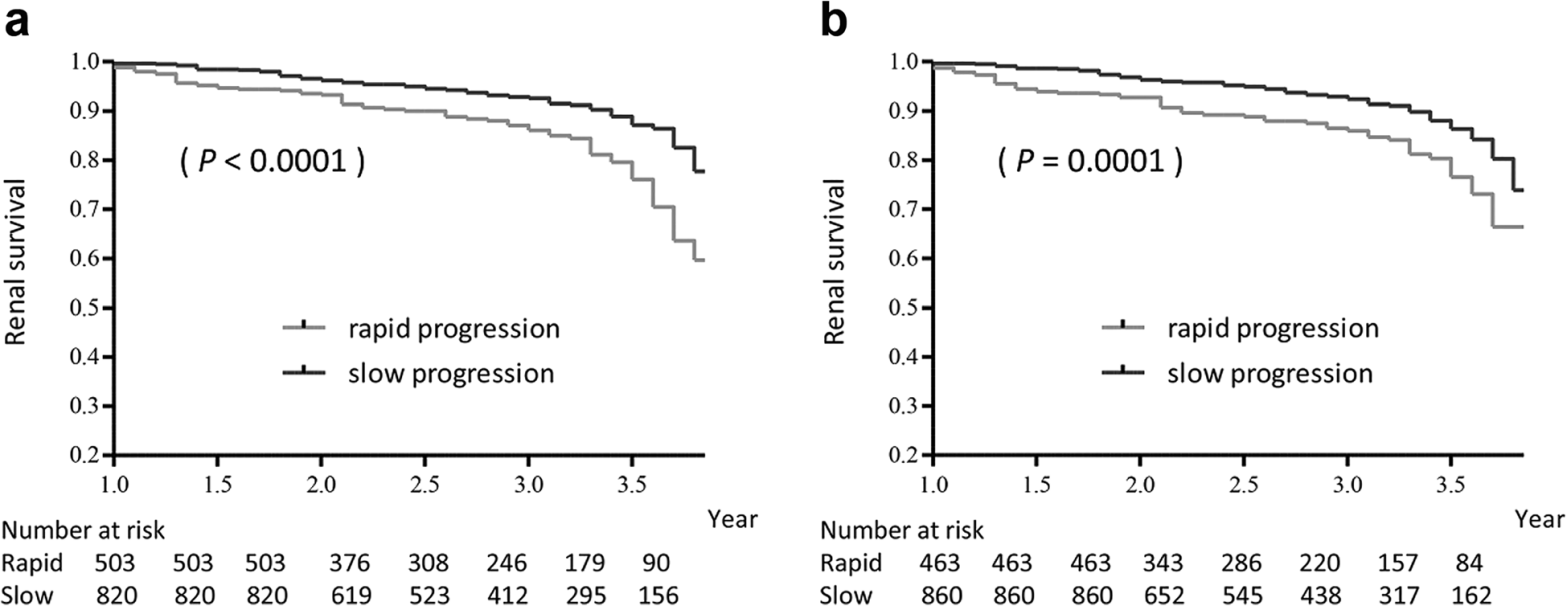
Cumulative renal survival according to rapid or slow progression evaluated in all the subjects. Cumulative end-stage renal disease (ESRD) risks were higher in the subjects with rapid progression than in those with slow progression based on serum creatinine (*p* < 0.0001) (**a**) and cystatin C levels (*p* = 0.0001) (**b**), respectively

During the follow-up period of 2.9 years (2.0–3.8 years), 134 subjects (10%) progressed to ESRD. We initially evaluated the association between rapid progression and renal outcome. When using the eGFRcr slope-based criteria, the cumulative ESRD risks were higher in the subjects with rapid progression than in those with slow progression (*p* <  0.0001, Fig. 2a). When using the eGFRcys slope-based criteria, the subjects with rapid progression were also associated with a higher incidence of ESRD than those with slow progression (*p* = 0.0001, Fig. 2b).

To adjust for the effect of baseline kidney function, we used Cox proportional hazard models (Table 2). Both the eGFRcr and eGFRcys slopes were associated with a higher risk of ESRD after adjustment for baseline eGFR (eGFRcr slope: HR = 0.986 [95% CI: 0.982–0.991], *p* <  0.0001; eGFRcys slope: HR = 0.988 [95% CI: 0.983–0.993], *p* <  0.0001). Both creatinine- and cystatin C-based rapid progressions were also associated with an increased risk of ESRD after adjustment for baseline eGFR (creatinine based-rapid progression: HR = 2.22 [95% CI: 1.57–3.13], *p* <  0.0001]; cystatin C-based rapid progression: HR = 2.03 [95% CI: 1.44–2.86], *p* <  0.0001).

**Table 2:** Effect of creatinine-based glomerular filtration rate slope on renal survival in comparison with that of cystatin C-based glomerular filtration rate slope: Adjusted for baseline estimated glomerular filtration rate

Variable	HR	95% CI	*P* value	*c-*index (95% CI)	*P* value
eGFRcr slope	0.986	0.982–0.991	< 0.0001	0.6425 (0.5912–0.6939)	0.239^b^
eGFRcys slope	0.988	0.983–0.993	< 0.0001	0.6309 (0.5796–0.6821)	
Creatinine-based rapid progression^a^	2.22	1.57–3.13	< 0.0001	0.6324 (0.5796–0.6852)	0.659^c^
Cystatin C-based rapid progression^a^	2.03	1.44–2.86	< 0.0001	0.6428 (0.5916–0.6941)	

eGFRcr-cys values were used for baseline eGFR values

*eGFR* Estimated glomerular filtration rate, *eGFRcr* Creatinine-based eGFR, *eGFRcys* Cystatin C-based eGFR, *HR* Hazard ratio, *95% CI* Confidence interval

^a^Rapid progression was defined as a eGFR slope steeper than −5 mL/min/1.73 m^2^/year

^b^*p* value was obtained by comparing the c-index of the eGFRcr slopes with that of the eGFRcys slopes

^c^*p* value was obtained by comparing the c-index of the creatinine-based rapid progression with that of the cystatin C-based rapid progression

**Fig. 3:**
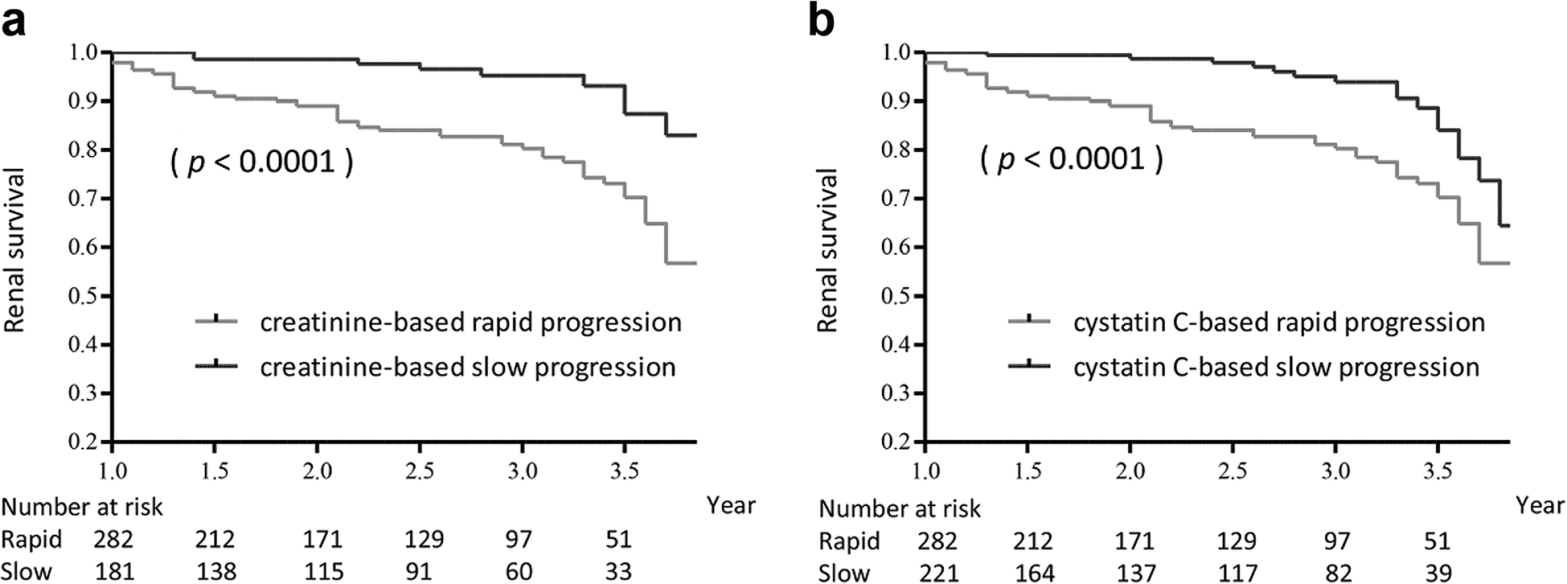
Cumulative renal survival according to rapid or slow progression evaluated by one marker in subjects with rapid progression stratified by the other marker. In the cystatin C-based rapid progression group (*n* = 463), the creatinine-based rapid progression group showed a higher incidence of end-stage renal disease (ESRD) than the creatinine-based slow progression group (*p* < 0.0001) (**a**). In the creatinine-based rapid progression group (*n* = 503), the cystatin C-based rapid progression group showed a higher incidence of ESRD than the cystatin C-based slow progression group (*p* < 0.0001) (**b**)

**Fig. 4:**
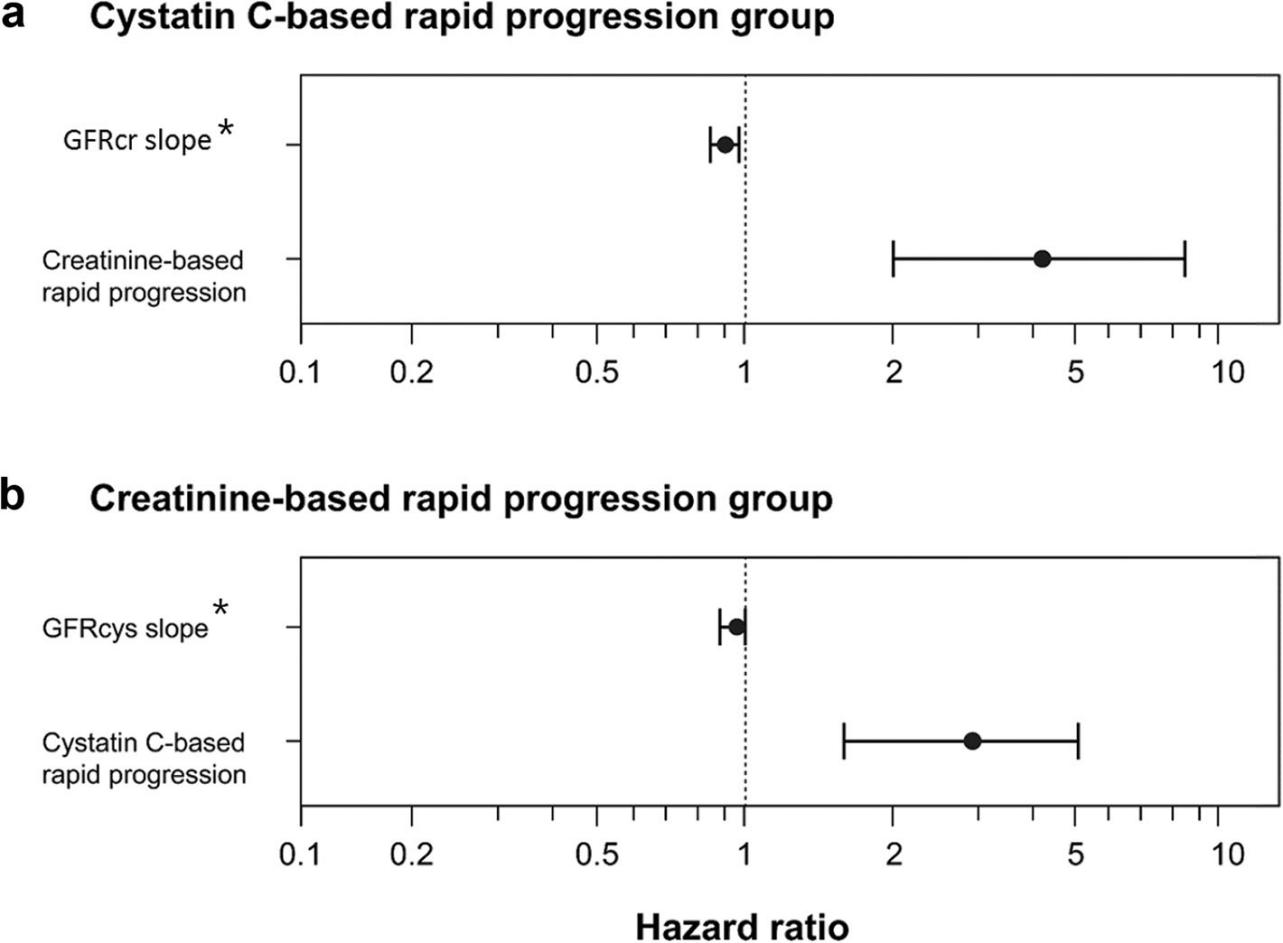
Adjusted hazard ratios (HR) of end-stage renal disease (ESRD) for eGFRcr and eGFRcys slopes among the rapid progression group stratified by either marker. After adjustment for baseline eGFR, both eGFRcr slopes and creatinine-based rapid progression were associated with higher risk of ESRD (eGFRcr slope: HR = 0.91 [95% confidence interval [CI]: 0.85–0.97], *p* = 0.003); creatinine-based rapid progression: HR = 4.25 [95% CI: 2.10–8.59], *p* < 0.001) (**a**). When adjusting for the baseline eGFR, the association between the eGFRcys slope and the risk of ESRD did not reach statistical significance (HR = 0.96 [95% CI: 0.88–1.04], *p* = 0.31) (**b**). *HR and 95% CI for 10 increments of eGFR slope (mL/min per 1.73 m^2^/year). eGFR, estimated glomerular filtration rate; eGFRcr, creatinine-based eGFR; eGFRcys, cystatin C-based eGFR

Next, we conducted subgroup analyses using the rapid progression group to evaluate the additional efficacy of combined use of these two markers. In the cystatin C-based rapid progression group (*n* = 463), the creatinine-based rapid progression group showed a higher incidence of ESRD than the creatinine-based slow progression group (20% vs. 5% at 3 years *p* <  0.0001, Fig. 3a). After adjustment for baseline eGFR, both eGFRcr slopes and creatinine-based rapid progression were associated with a higher risk of ESRD (eGFRcr slope: HR = 0.91 [95% CI: 0.85–0.97], *p* = 0.003; creatinine-based rapid progression: HR = 4.25 [95% CI: 2.10–8.59], *p* < 0.001; Fig. 4a). Within the creatinine-based rapid progression group (*n* = 503), the cystatin C-based rapid progression group showed a higher incidence of ESRD than did the cystatin C-based slow progression group (*p* < 0.0001, Fig. 3b). However, when adjusting for baseline eGFR, the association between eGFRcys slope and the risk of ESRD did not achieve statistical significance (HR = 0.96 [95% CI: 0.88–1.04], *p* = 0.31; Fig. 4b).

## Discussion

In the present study, we compared the prognostic powers of the eGFRcr and eGFRcys slopes determined by using serial measurements of both markers over a 1-year period in outpatients with stage 3 to 5 CKD. eGFRcr slopes were associated with an increased risk of ESRD in patients with CKD, independently of baseline kidney function. In addition, the eGFRcr slope allowed further discrimination of higher risk of ESRD in the rapid progression group, defined on the basis of cystatin C level. The eGFRcys slope was also associated with an overall risk of ESRD. However, the association between eGFRcys slope and the risk of ESRD was not evident in patients who met the criteria for rapid progression based on serum creatinine levels. Previous studies on the performance of creatinine or cystatin C level focused on its cross-sectional aspect, specifically renal function at the point of assessment [20]. This study compared the usefulness of the eGFRcr and eGFRcys trends to predict progression to ESRD in patients with CKD and determined that the eGFRcr slope was not different to the eGFRcys slope.

Many studies have shown that an early change in eGFR predicts the risk of CKD progression using serum eGFRcr. A ≥ 25% decrease in eGFR over 1 year is associated with increased risk of ESRD [5]. The early slope of eGFR decline in patients with type 1 diabetes can be used to predict the risk of ESRD [7]. Several studies have been conducted on the longitudinal behavior of cystatin C level. Perkins et al. [25]. first mentioned the usefulness of serial measurement of serum cystatin C level to accurately detect trends in renal function in 30 diabetic subjects with normal or elevated GFR. Shlipak et al. [26] demonstrated that declining kidney function based on cystatin C levels is associated with a higher risk of heart failure, myocardial infarction, and peripheral artery disease among patients with or without CKD. Premaratne et al. [27] showed that the change in eGFRcys correlated well with changes in measured GFR than with changes in eGFRcr in type 1 diabetes with preserved kidney function, suggesting that cystatin C levels are more sensitive for the detection of an early decline in GFR in patients with normal kidney function. By contrast, this study demonstrated that the eGFRcr slope was not different to the eGFRcys slope for the assessment of kidney function in patients already exhibiting decreased kidney function.

A few studies have reported that serum cystatin C level has a somewhat weaker correlation with measured GFR than serum creatinine [28]. Rule et al. [29] showed that the biases of the cystatin C-based estimating equation differed for specific patient groups such as those with native kidney disease, kidney transplant recipients, and potential kidney donors, suggesting that non-GFR determinants impact serum cystatin C levels considerably. The research group [30] also demonstrated that the reliability (mean coefficient of variation) of eGFRcr (6.4%) is better than that of eGFRcys (10.7%) in a large community-based cohort. Both test reliability and non-GFR determinants might have affected the accuracy of eGFR slopes in our study. Serum creatinine level seemed to be less vulnerable to both factors than serum cystatin C levels. Nevertheless, a growing body of evidence suggests that serum cystatin C levels are good predictors of all-cause mortalities [31–33]; thus, non-GFR determinants of serum cystatin C level may overlap conventional risk factors of cardiovascular diseases such as diabetes, chronic inflammation, obesity, and lower serum albumin level [34, 35].

Implementing the evaluation of a creatinine-based eGFR slope in clinical practice might have advantages to cystatin C-based eGFR in terms of cost. The unit costs of cystatin C testing are 6 times higher than that of creatinine in Korea. Given that sequential measurements are needed to track changes in kidney function, the costs involved for measurements should be considered. A recent study that compared creatinine with cystatin C levels has also discussed the cost-effectiveness of both markers [36]. Considering that the eGFRcr slope was not different to the eGFRcys slope in predicting CKD progression in our study, the use of creatinine level should be considered more cost effective than that of cystatin C level when tracking the renal function in patients with CKD.

This study has several limitations. First, as a retrospective study, it may have some selection bias. However, this study collected laboratory data mainly from electronic medical records, and both filtration markers were measured simultaneously; thus, the chances of measurement error or misclassification were fewer. Considering that large sample numbers are needed to assess progression to ESRD as a primary outcome, our findings based on retrospective data are worthy of attention. Second, serum creatinine levels were measured using the Roche enzymatic method, in agreement with the CKD-EPI equation development study [22]; however, serum cystatin C levels were measured using the Gentian cystatin C assay at our center. Nevertheless, Flodin et al. confirmed that the latter assay has good agreement with the data obtained from the Dade Baring Nephelometer assay used in the CKD-EPI equation development study [37]. Several studies have shown reliable results using various cystatin C-based equations, including the CKD-EPI equation based on the Gentian cystatin C assay [38–40]. Although a certified reference material, ERM-DA471/IFCC, is available for cystatin C, there were substantial variability in cystatin C values according to the commercially available measurement procedures [41]. Thus, a different assay used for cystatin C measurements would have likely influenced the study results. Finally, our study population consisted of Asian subjects who were less obese than those in other studies that evaluated CKD. Thus, the generalizability of our findings remains to be determined. Further studies are needed to examine the validity of these findings in other populations.

## Conclusion

This study investigated the prognostic efficacy of eGFR slopes calculated by simultaneous measurement of two endogenous filtration markers, creatinine and cystatin C levels, over a 1-year period in patients with decreased kidney function. Both eGFR slopes were effective in identifying subjects at a higher risk of ESRD, and the eGFRcr slope was not different to the eGFRcys slope as a predictor of renal outcome in patients with CKD. Although serum cystatin C level has been reported to be a better predictor of cardiovascular disease and mortality than serum creatinine level, the latter may reflect GFR trends more directly, which may be useful for proper management of renal replacement therapy.

## Data Availability

The datasets used and/or analysed during the current study are available from the corresponding author on reasonable request.

## Abbreviations

ACE-I: Angiotensin-converting enzyme inhibitor
ACR: Albumin-to-creatinine ratio
ARB: Angiotensin receptor blocker
BMI: Body mass index
CI: Confidence intervals
CKD: Chronic kidney disease
CKD-EPI: Chronic Kidney Disease Epidemiology Collaboration
CRP: C-reactive protein
DM: Diabetes mellitus
eGFR: Estimated glomerular filtration rate
eGFRcr: Creatinine-based estimated glomerular filtration rate
eGFRcys: Cystatin C-based estimated glomerular filtration rate
ESRD: End-stage renal disease
GN: Glomerulonephritis
HR: Hazard ratio
IQR: Interquartile range
IRB: Institutional review board
KT: Kidney transplantation
PCKD: Polycystic kidney disease
PCR: Protein-to-creatinine ratio
SD: Standard deviation
